# Evaluation of the Inclusion of the Green Seaweed *Ulva ohnoi* as an Ingredient in Feeds for Gilthead Sea Bream (*Sparus aurata*) and European Sea Bass (*Dicentrarchus labrax*)

**DOI:** 10.3390/ani11061684

**Published:** 2021-06-05

**Authors:** Francisca P. Martínez-Antequera, Juan A. Martos-Sitcha, Jose M. Reyna, Francisco J. Moyano

**Affiliations:** 1 Department of Biology and Geology, Faculty of Experimental Sciences, Campus de Excelencia Internacional del Mar (CEI·MAR), University of Almería, 04120 Almería, Spain; fjmoyano@ual.es; 2 Department of Biology, Faculty of Marine and Environmental Sciences, Instituto Universitario de Investigación Marina (INMAR), Campus de Excelencia Internacional del Mar (CEI·MAR), University of Cádiz, 11519 Cádiz, Spain; inmar@uca.es; 3 CTAQUA Centro Tecnológico de Acuicultura, Commercial Dock S/N, 11500 Cádiz, Spain; info@ctaqua.es

**Keywords:** aquaculture feeds, bioactive compounds, *Ulva ohnoi*

## Abstract

**Simple Summary:**

The use of seaweeds in aquafeeds is receiving increasing attention due to their potential nutritional and functional benefits. However, several green seaweeds such as *Ulva* presents nutritional limitations because of the undigestible polysaccharides, although these may exert a positive effect on the immunological status of the fish. The present study developed three different experiments aimed to re-evaluate the presence of protease inhibitors described for *Ulva ohnoi*, to assess its nutritional value as an ingredient and also to evaluate its potential protective effect on the oxidative metabolism of fish, being experiments developed in two different fish species (European sea bass *Dicentrarchus labrax* and gilthead sea bream, *Sparus aurata*). Results indicate the absence of negative effects of *U. ohnoi* on protein digestion of sea bream but a limited value as a feed ingredient. In contrast, its contents in bioactives seem to be correlated to the observed positive effects on the immune status and oxidative metabolism when fish are challenged by the consumption of highly oxidized dietary oil.

**Abstract:**

This study evaluated the use of *Ulva ohnoi* as an ingredient in feeds for aquaculture in three different experiments. Experiment 1 was oriented to confirm the negative effect of *U. ohnoi* on fish digestion. Experiment 2 assessed the effect on growth, feed efficiency, and immune status of juvenile sea bass (*Dicentrarchus labrax*) fed on diets including *U. ohnoi*, previously treated or not with carbohydrases used to partially hydrolyze indigestible polysaccharides. Experiment 3 was aimed to evaluate the potential protective effect of *U. ohnoi* on the oxidative status of sea bream (*Sparus aurata*) challenged by the consumption of a feed formulated with the oil fraction completely oxidized. Results show a negligible effect of *U. ohnoi* meal on protein digestion when included in feeds at levels of 10% or less. Moreover, results of growth and feed use evidenced the possibility of using up to 5% inclusion of algal meal in feeds without adverse effects on the zootechnical parameters, while the enzyme pretreatment was ineffective to improve its nutritional use. Finally, the inclusion of *U. onhoi* in feeds determined both an immunostimulatory effect, evidenced by an increase in skin mucus lysozyme in the two mentioned fish species, and a positive influence on the oxidative metabolism of seabream when fed on a diet including rancid oil.

## 1. Introduction

*Ulva* are green macroalgae belonging to the phylum Chlorophyta that presents a great environmental polymorphism, and genetic analysis suggests that the different described species for the genus (*U. armoricana, rigida, prolifera, pertusa, fasciata,* or *ohnoi*) are only environmental variants or clades [1]. *Ulva* blooms are frequent and described in several parts of the world associated with an excess of dissolved nutrients in coastal waters resulting from fields fertilization or human wastes [2]. The potential use of *Ulva* biomass provided by these blooms as a source of fertilizer or bioenergy has been widely assessed [3]. Several studies have tested the potential inclusion of *Ulva* in aquaculture feeds, although previous results pointed that the observed effects are closely related to the level of dietary inclusion. Hence, positive effects on growth and feed efficiency have been reported at incorporation rates accounting for less than 10% of the dry weight of the feed [4,5]. In contrast, levels exceeding this amount either do not result in significant effects [6,7,8] or produce negative results [9,10]. In this sense, it has been suggested that the presence of protease inhibitors may limit digestive use of this seaweed in several fish species such as the Senegalese sole (*Solea senegalensis),* the gilthead seabream *(Sparus aurata),* or the European sea bass *(Dicentrarchus labrax*,) and hence limiting growth performance and feed use [11].

On the other hand, the role of seaweeds as sources of bioactive compounds is widely recognized [12,13]. In the case of *Ulva* species, the polysaccharide ulvan may present beneficial effects on the immunological status in some fish species such as *Solea senegalensis* [14], and they also contain compounds with reported antioxidant effect [15,16,17]. Nevertheless, results are somewhat contradictory, and some authors did not find such positive effects when including this seaweed in diets for marine species such as *D. labrax* [18] or *S. senegalensis* [19]. For this reason, the possibility of testing the potential positive effect of using *Ulva* as a protective agent against conditions determining oxidative stress was considered within the framework of this general evaluation. The selected challenge was the consumption of rancid oil since this has been reported as one factor with a high impact on the growth and oxidative metabolism of fish [20,21,22].

Considering all the aforementioned information, it is clear that the potential inclusion of seaweeds in fish feeds must consider different aspects related to their potential role, either as nutritional or functional ingredients. To date, no comprehensive study has assessed the potential benefits of including *Ulva* in fish feeds from both perspectives. The present work was intended to evaluate biological responses obtained when using *Ulva ohnoi* as an ingredient in feeds for two important marine fish for European aquaculture, the European sea bass (*D. labrax*) and the gilthead seabream (*S. aurata*). The study developed different experiments focused on the evaluation of nutritional aspects by using both in vitro and in vivo approaches, as well as the potential benefits derived from the presence of bioactive compounds on immunological parameters and oxidative status of fish.

## 2. Materials and Methods

### 2.1. Algal Biomass

The biomass of *U. ohnoi* used in the different experiments was collected from external tanks of the facilities of the Aquaculture Technology Center CTAQUA (El Puerto de Santa María, Spain). After washing with fresh water, the biomass was partially desiccated using a solar dryer until it reached a moisture content of nearly 20%. Once received in the laboratory, the biomass was subjected to an additional drying in an oven for 24 h at 60 °C and subsequently finely chopped until obtaining a fine powder that was used in all the assays.

### 2.2. Description of the Experiments

**Figure 1:**
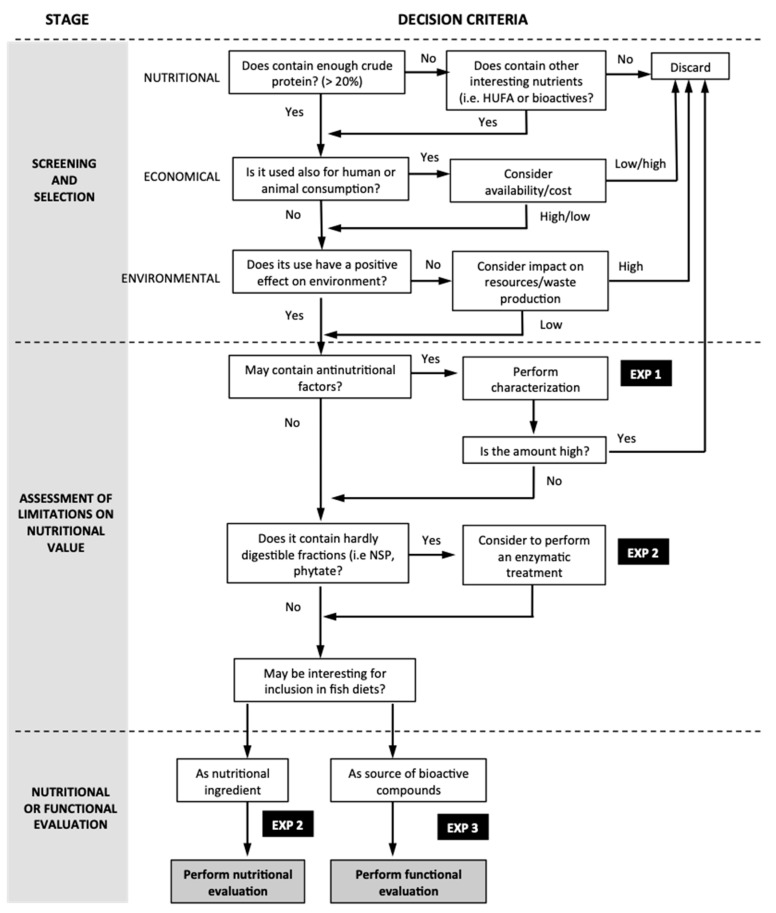
Flow diagram used to design the experiments developed in the present study.

As indicated in the previous section, 3 different experiments were designed based on the flow diagram and decision criteria detailed in Figure 1 and described below.

### 2.3. Experiment 1

This preliminary assay was oriented to confirm the potential negative effect of *U. ohnoi* on fish digestion.

According to the study of Vizcaíno et al. (2020) [11], *U. ohnoi* contains a protease inhibitor of fish digestive proteases that would inactivate up to 60% of the alkaline protease activity present in the gut of different fish species such as European sea bass or Senegalese sole. However, such a study did not consider the amounts of algal meal and enzyme that could be really present in the digestive tract of fish consuming a feed enriched with the seaweed. The present experiment was designed to quantify on a physiological basis such expected inhibition by; (a) developing an inhibition assay after establishing accurately the expected relationship between the amount of algae and enzyme activity present in the digestive system of juvenile seabass fed on a standard ration, and (b) using an in vitro assay to confirm the effect of the protease inhibitor on the digestive hydrolysis of protein present in the feed.

To develop point (a), the total amount of protease activity in the gut of juvenile sea bass during digestion was measured on fish sampled 3 h after being fed on a commercial feed (*n* = 10; 40.6 ± 2.8 g). Digestive enzyme extracts were prepared after manual homogenization of dissected tissues, and the determination of acid and alkaline protease activities was carried out using hemoglobin and casein as substrates, respectively [23,24]. On the other hand, the expected amount of *Ulva* present in the digestive tract was estimated considering the average amount of feed consumed per meal in a fish of the above-indicated size and an inclusion level of *Ulva* in the feed of 80 g/kg. Both data (total activity produced by a fish and total amount of algae ingested per meal) were used to determine the % inhibition of protease activity potentially derived by using such amount of algae. The inhibition assay was carried out similarly to that described by Vizcaíno et al. (2020) [11] by mixing 1 mL of intestinal enzyme extract of known activity to the required amount of seaweed meal to achieve the E:S ratio calculated above. The mixture was incubated for 60 min, and the observed reduction in activity was expressed as % of that determined using an extract preincubated only in the presence of water as a reference.

In a second approach, the potential effect of protease inhibitor on digestive protein hydrolysis was estimated more precisely by an in vitro digestive simulation test. The assay was developed using the membrane bioreactor and general procedure described in Gilannejad et al. (2017) [25]. In short, the device consists of two chambers separated by a semi-permeable membrane of 3500 kDa MWCO (ZelluTrans/Roth1). Enzyme extracts and feed samples were placed in the upper chamber and maintained under continuous agitation using a magnetic stirrer. Amino acids passing across the membrane into the lower chamber were recovered at different time intervals during the reaction time. During the acid phase of digestion, the upper chamber contained the substrate dissolved in water and adjusted to pH 4.0 as well as the crude enzyme extract from the seabass stomach, while the lower chamber contains distilled water. During the alkaline phase, the pH of the upper chamber was raised to pH 8.5 prior to the addition of the intestinal enzyme extracts, being the lower chamber filled with 100 mM Tris-maleate buffer at the same pH (supplemented with 100 mM CaCl_2_ and 50 mM NaCl). The complete arrangement was maintained at 25 °C. Total amino acids released during the hydrolysis were measured using the o-phtaldialdehyde method [26]. The assay developed using this configuration tested differences in protein hydrolysis obtained using the E:S ratio described above with samples of feeds C (without *Ulva* meal) and U8 (containing 80 g/kg *Ulva*) formulated for Experiment 2 (Table 1).

### 2.4. Experiment 2

This experiment was oriented to assess the effect on growth, feed efficiency, and immune status of juvenile sea bass fed on diets including *Ulva*, previously treated or not with a mixture of carbohydrases used to partially hydrolyze the fraction of indigestible polysaccharides.

#### 2.4.1. Enzyme Pretreatment of Ulva

**Figure 2:**
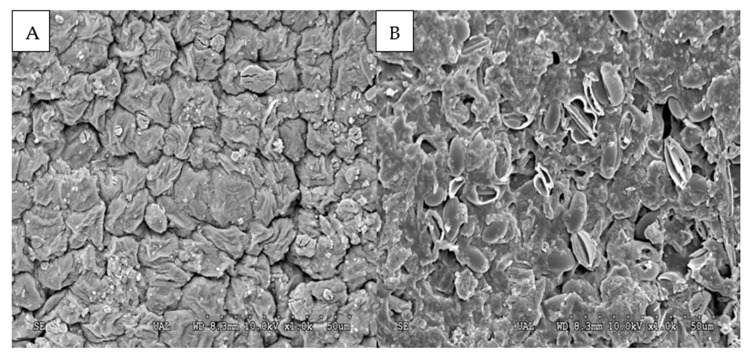
Scanning electron microscopy of a sample of the meal of *Ulva ohnoi* (**A**) before enzymatic hydrolysis and (**B**) after 24 h of enzymatic hydrolysis.

A certain amount of the *Ulva* meal described in Section 2.1. was enzymatically pre-treated with a commercial mixture of carbohydrases (Rovabio Advance Max L), which presented a high activity of glucanases and pectinase. To carry out the treatment, the meal was mixed carefully with citrate buffer (pH 5.0, 0.1 M; 1:3 w/v) to obtain a moist mass with the optimal conditions for the action of the enzymes, which were previously solubilized in a small amount of the same buffer and added by spraying. The enzyme mixture was added using the dose indicated by the producer (0.2 mL/kg) and allowed to act by keeping the mixture at 45 °C for 6 h, with manual stirring every hour to ensure the homogeneity of the reaction. After this time, the reaction was stopped by placing the mixture in a cold chamber at 4 °C until being used as an ingredient in the preparation of the experimental diets. Besides the chemical analysis described in the next section, scanning electron microscopy was used to assess the potential effect of enzyme treatment on the tissue structure of *Ulva*. Several images were obtained from different areas of the samples to guarantee the representativeness of the results (Figure 2).

#### 2.4.2. Diet and Analysis

Five experimental feeds were formulated, including either 5% or 8% of *Ulva* meal, enzymatically treated (UE5, UE8) or not (U5, U8). A diet without alga meal was used as a control (C) (Table 1). Cr_2_O_3_ was included in all diets as an inert marker to evaluate digestibility. Feeds were prepared using a lab-scale extrusion machine provided with a mesh size of 2 mm, dried, and stored at 4 °C until used. Besides proximate analysis, samples of each feed were used for the analysis of some compounds that could be affected by the enzyme pretreatment, as soluble protein, reducing sugars, free pentoses, total phenolics, and total antioxidant capacity. Soluble protein was analyzed by the Bradford method (1976) [27] using the SIGMA Total Protein Kit (TP0100). Reducing sugars were measured using 3,5-dinitrosalicylic acid (DNS) following the method described by Miller (1959) [28]. Free pentoses were measured by the phloroglucinol method described by Douglas (1981) [29]. Total phenolics were determined by the method described by Graça et al. (2005) [30]. Trolox equivalent antioxidant capacity (TEAC) was determined following the DPPH method described by Brand-Williams et al. (1995) [31]. All the analyses were performed in triplicate samples from each diet.

#### 2.4.3. Animals and Facilities

A total of 450 juvenile European sea bass (*Dicentrarchus labrax*) (16.4 ± 1.2 g) were distributed into 15 tanks (120 L; *n* = 30 fish per tank) in the facilities of CTAQUA (El Puerto de Santa María, Spain). The tanks were provided with a settling column for stool removal (Guelph method). Each of the five experimental feeds was evaluated in triplicate. Each experimental diet was offered to visual satiety two times per day and 6 days per week in a 67-day feeding trial. The amount of food ingested by each experimental unit was recorded on a weekly basis using gravimetric methods (g of feed consumed / tank). Water quality of the system was continuously monitored; temperature, dissolved oxygen, and survival data were controlled daily, whereas ammonium, nitrite, and salinity levels were checked weekly. For the digestibility assay, feces were removed daily for 3 weeks, dried, and processed to determine their nitrogen contents. Fecal samples obtained on three different days were pooled to form one sample, and three different samples were obtained from each tank. The determination of the total chromium of feeds and feces was carried out using the diphenylcarbazide method [32].

#### 2.4.4. Parameters Evaluated

The following growth parameters were evaluated:

(1) specific growth rate (SGR) = (100 × (ln final body weight—ln initial body weight))/days
(2) weight gain (WG) = (100 × (body weigh increase))/initial body weight
(3) feed conversion ratio (FCR) = total feed intake/weight gain.

Moreover, apparent digestibility coefficients (ADC) were calculated as follows:

(1) $$ADC\;nutrient=100-\left[⟦\frac{\left(\%\;\mathrm{of}\;\mathrm{indicator}\;\mathrm{in}\;\mathrm{food}\right)}{\left(\%\;\mathrm{of}\;\mathrm{indicator}\;\mathrm{in}\;\mathrm{feces}\right)}⟧⟦\frac{\left(\%\;\mathrm{of}\;\mathrm{nutrient}\;\mathrm{in}\;\mathrm{feces}\right)}{\left(\%\;\mathrm{of}\;\mathrm{nutrient}\;\mathrm{in}\;\mathrm{food}\right)}⟧*100\right]$$

The potential variations in the immunological status of the fish after being fed on diets including *Ulva* were assessed by measuring lysozyme and alkaline phosphatase activities in skin mucus (12 fish per diet) sampled at the end of the growth experiment. Lysozyme was measured using a commercial kit (Ref. E22013; Thermo Fisher Scientific, Waltham, Massachusetts, USA), adapted to 96-well microplates. Alkaline phosphatase activity was determined using pNp-phosphate disodium salt (Sigma-Aldrich M8168) as substrate following the method described by Gee et al. (1999) [33].

### 2.5. Experiment 3

This experiment was aimed to evaluate the potential protective effect of *Ulva* on the oxidative status of fish challenged by the consumption of a feed including oxidized oil.

#### 2.5.1. Ingredients and Feeds

The *U. ohnoi* meal was the same as in previous experiments. Four experimental feeds were elaborated (Table 2); two of them included 10% of *U. ohnoi* meal and either fresh or oxidized oil (U/UO). The other two control feeds followed the same scheme and also included the two types of oil (C/CO). Oxidation of the mixture of fish and sunflower oils used in the elaboration of CO and UO feeds was produced by heating at 60 °C with intermittent air injection (10 min of injection and 30 min of rest) for 24 h until the peroxide value (POV) reached 101.25 meq/kg (the value for untreated oil was 0.60 meq/kg). Feeds were prepared using a lab-scale extrusion machine provided with a mesh size of 2 mm, dried, and stored at 4 °C until used. Samples of each feed were used for the analysis of soluble protein, reducing sugars, free pentoses, total phenolics, and antioxidant capacity, according to the methodologies described in Section 2.4.

#### 2.5.2. Animals and Feeding Schedule

A total of 400 juvenile gilthead sea bream (*Sparus aurata*) (46.31 ± 0.29 g) were distributed into 12 tanks (330 L; *n* = 32 fish per tank) in the facilities of CTAQUA (El Puerto de Santa María, Spain). The water quality of the system was continuously monitored as described in the previous section. Each of the four experimental feeds was evaluated in triplicate. Each experimental diet was offered to visual satiety two times per day and 6 days per week. The amount of feed ingested by each of the experimental units was recorded on a weekly basis using gravimetric methods (g of feed consumed/tank). The trial ran for 28 days, being this period divided into three stages: preliminary feeding (7 days), challenge (14 days), and recovery (7 days). During the preliminary feeding, all the fish were fed on a commercial feed to normalize their nutritional status. During the challenge, triplicate groups of 30 fish received each of the 4 types of experimental feeds. After this period, during recovery, the fish groups that were fed on feeds containing rancid oil (CO and UO) received the feed containing *Ulva* (U), while the other two groups maintained the same feeds consumed during the previous stage.

#### 2.5.3. Evaluated Parameters

At the end of the trial, overnight fasted fish (5 fish per tank, 15 per experimental condition) were randomly sampled and anesthetized with 2-fenoxiethanol for liver and skin mucus collection. Previously, fish were bled out with heparinized syringes and killed by the cervical section, and their livers were extracted and weighed to calculate the hepatosomatic index (IHS). Samples of liver and skin mucus were rapidly taken, snap-frozen in liquid nitrogen, and stored at −80 °C until used in biochemical analyses.

Livers were homogenized (1:10, w/v) in 100 mM potassium phosphate buffer (pH 7.4) at 4 °C using a mini handheld homogenizer (Ref. MT-13K; Hangzhou Miu Instruments Co., Ltd., Hangzhou, China) for 1 min. Homogenates were centrifuged at 12,000× g for 15 min at 4 °C, and supernatants were used to determine different enzyme activities: superoxide dismutase (SOD), catalase (CAT), and glutathione peroxidase (GPx). Superoxide dismutase was measured using the commercial kit (Ref. CS0009; Sigma-Aldrich, St. Louis, MO, USA). Catalase was measured using a commercial kit (Ref. EIACATC; Thermo Fisher Scientific, Waltham, MA, USA). Glutathione peroxidase was measured using a commercial kit (Ref. 703102; Cayman Chemical, Ann Arbor, MI, USA). Lipid peroxidation was assessed by measuring total thiobarbituric acid reactive substances (TBARS) using the method of Buege and Aust (1978) [34]. In addition, mucus lysozyme was measured (5 fish/tank) as described in Section 2.4.

### 2.6. Statistical Analysis

The normality of the data was performed using the Shapiro–Wilk test, and homoscedasticity analysis was conducted using the Brown–Forsythe test. Statistical analysis of the data was carried out by one or two-way ANOVA, followed by the Bonferroni test where appropriate. The significance level was established at *p* < 0.05. When required, data expressed in percentage were previously arc-sin transformed. All the analyses were performed using the software Statgraphics Centurion (Statgraphics Corp. CA. EE.UU.).

## 3. Results

### 3.1. Inhibitory Effect of U. ohnoi Meal on Protein Digestion in European Sea Bass

**Figure 3:**
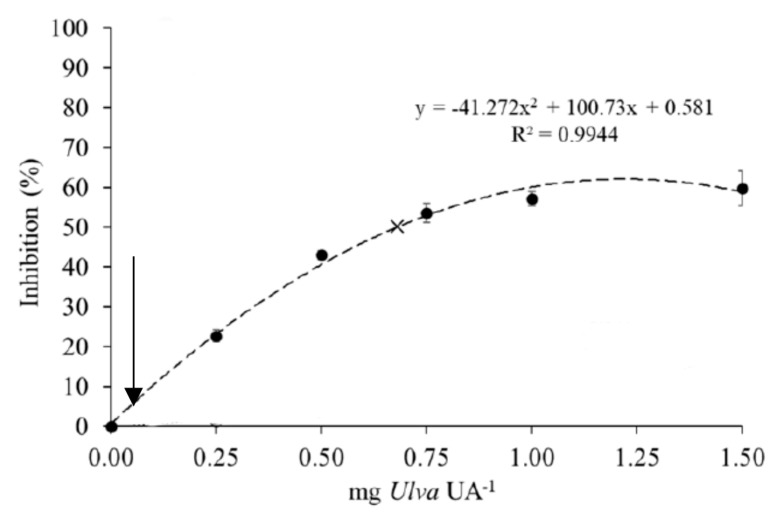
Inhibitory response of European sea bass intestinal proteases after incubation with increasing doses of raw or heat-treated *Ulva* extracts (graph adapted from Vizcaíno et al., 2020) [11]. The inhibition value (% over a control) corresponding to 0.02 mg *Ulva*/U activity is represented by an arrow.

**Figure 4:**
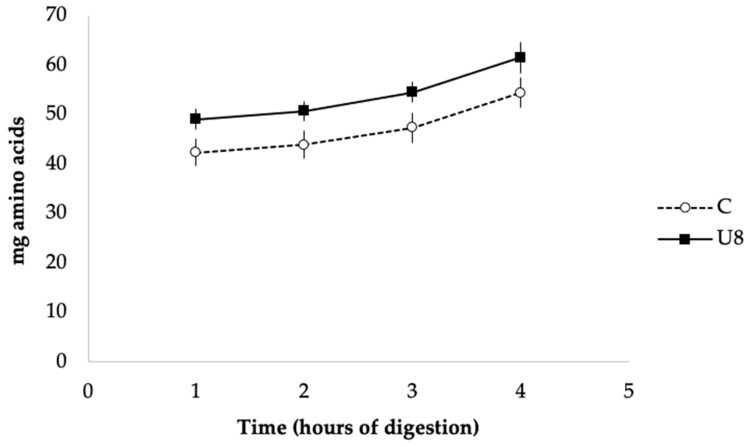
Time release of total amino acids in the in vitro simulation of the digestion of feed, including 8% *U. ohnoi* meal (U8) and without macroalgae (C). Each point represents the average value of three determinations ± SD.

The activity of digestive alkaline proteases measured in juvenile sea bass was 62 U/g fish. Accordingly, a 40 g fish (representative size for a juvenile fish) should produce about 2500 U of enzyme in each feeding episode. On the other hand, a fish of such size receives two meals daily (1.5% of the weight/meal) this accounting for 0.6 g feed/meal. If such feed contains 80 g/kg of *U.* *ohnoi* meal (an average amount estimated from the studies cited in the Introduction section), the estimated intake of seaweed per meal should be around 50 mg/meal. This should result in a relative proportion of 0.02 mg *U. ohnoi* per unit enzyme activity in each feeding episode. When such value is represented in the plot published by Vizcaíno et al. (2020) [11], it results in less than 10% protease inhibition (Figure 3). This was coincident with the result obtained in the inhibition assay, which produced a 10.7% decrease in the activity of sea bass alkaline proteases. This negligible effect was confirmed by results obtained with the in vitro assay, on which no visible reduction in the hydrolysis of the protein fraction associated with the presence of *U. ohnoi* meal in the feed was appreciated (Figure 4).

### 3.2. Effect on Nutritional Efficiency and Immune Status of Juvenile European Sea Bass Fed on Diets Including U. ohnoi Previously Treated or Not with a Mixture of Carbohydrases

The potential differences in the amounts of some readily bioavailable nutritional compounds (soluble protein, reducing sugars, pentoses) or bioactives (total phenols, TEAC) in the different experimental diets are detailed in Table 3. Results evidenced a significant reduction in the amount of soluble protein in diets including *Ulva* when compared to the control, as well as a negative effect of the enzyme treatment on this parameter. The amount of reducing sugars was also significantly lower in feeds containing *Ulva* meal when compared to the control diet, although no effect of the enzyme treatment was observed in this case. In addition, neither the presence of *Ulva* nor the enzyme treatment influenced the amount of pentoses or total phenols, while TEAC was significantly increased in feeds containing *U. ohnoi* in relation to the control.

Results regarding growth performance and feed use obtained when providing the experimental feeds for 65 days to juvenile European seabass are summarized in Table 4. During the experimental period, fish grew adequately and multiplied their weight by a factor of ~2.45, with an overall SGR ~1.3–1.4% day^−1^. There were no significant mortalities, and they maintained healthy and active. Overall, the results were quite homogeneous, and no clear effect of the inclusion of *Ulva* meal, treated or not, was evidenced. No significant differences were found in FCR between groups, but they were present in growth rates, with significantly lower rates obtained for fish fed on the feeds, including the higher amount of *U. ohnoi* meal, irrespective of enzyme treatment. Values of apparent digestibility for protein were significantly higher for these same diets and also for the diet, including the lower amount of algal meal enzymatically treated.

Values of the mucosal enzymes measured at the end of the growth period as indicators of the immune status of fish fed on the control diet and those including untreated *Ulva* meal are resumed in Table 5. Significantly higher values of both lysozyme and alkaline phosphatase were measured in fish receiving the higher amount of *U. ohnoi*.

### 3.3. Experiment 3. Evaluation of the Effect of Ulva on the Oxidative Status of Fish Challenged by the Consumption of a Feed Including Rancid Oil

As in the previous experiment, the potential differences in some bioactive compounds between the diets were evaluated by measuring total phenols and TEAC (Table 6). While the content of total phenols was not affected by the addition of macroalgae, these feeds presented a slight but significant increase in total antioxidant capacity. Although this was not a growth experiment, weight changes were also monitored in order to assess possible effects on the nutritional status of the fish. No significant differences in weight were observed between groups either after the challenge or the recovery periods. In addition, no mortality was recorded during the whole experiment. Values of the hepatosomatic index (HIS) measured at the end of the challenge showed significantly lower values in fish receiving feeds containing *Ulva*, regardless they included rancid oil or not, and the general trends were maintained after the recovery period (Table 7).

The immunological and oxidative status of the fish is detailed in Table 8. After the challenge, only fish fed the control diet, including rancid oil, showed a significantly lower value of mucus lysozyme. After the recovery period, these same fish evidenced a significant increase in the activity of lysozyme while the rest of the groups showed homogeneous values. In addition, the oxidative status of fish measured through the activities of different enzymes present in the liver evidenced some clear differences between the experimental groups. While no significant differences in the activity of SOD were observed between groups either during the challenge or after recovery, the activity of GPx was significantly lower in fish receiving feeds, including *Ulva*, irrespective if they included rancid oil or not. On the other hand, no effect was observed after the feed change in any of the two groups (C or U) that initially were fed on feeds, including rancid oil. In the case of CAT, high values were measured in all groups after the challenge, with the exception of fish receiving the control diet with fresh oil that showed a significantly lower value. Nevertheless, values of this enzyme measured after recovery showed a different pattern, with higher values observed in fish fed any of the diets, including *Ulva,* when compared with their equivalents in control fish. Moreover, fish initially fed on any of the diets, including rancid oil (UO, CO), showed a significant reduction in the values during the recovery period. Lipid peroxidation measured as MDA was evidenced by significantly higher values in fish consuming rancid oil in any of the two feeds (C or U) during the initial challenge period. After the recovery, only those fish initially fed on *Ulva* + rancid oil (UO) showed a significant decrease in lipid peroxidation, while the effect was not evident in those that received the control + rancid oil (CO).

## 4. Discussion

### 4.1. Inhibitory Effect of U. Ohnoi Meal on Protein Digestion in European Sea Bass

A number of papers evaluate the potential inhibitory effect of some ingredients on the digestive proteases of different species of aquatic animals. These studies, developed using a wide range of relative concentrations of both the enzymes and the potential inhibitors, provide useful information on the sensitivity of such enzymes on a species-specific basis [35,36]. Nevertheless, results must be carefully interpreted considering the real physiological conditions existing in the gut of the organisms. When doing this, as in the present study, an almost negligible effect was evidenced, and values of 60% inhibition, as those reported for proteases of marine fish by Vizcaíno et al. (2020) [11], appear to be out of range. Moreover, instead of the presence of a Bowman-Birk protein inhibitor suggested in such study, the effects should be better interpreted as a result of the interaction between digestive enzymes and polyphenols present in *Ulva* species, that may reach 75 mg/100 g [16] and which negative interactions have been previously reported [37,38]. On the other hand, the closer simulation of the digestion process performed in the present study, including the acid stage, resulted in no effect on the hydrolysis of feed protein in the presence of *U. ohnoi* meal, suggesting that a similar response could be obtained in vivo. Results attained in Experiment 2 regarding the digestibility of protein in feeds, including *U. ohnoi* meal, confirmed this hypothesis.

### 4.2. Effect on Nutritional Efficiency and Immune Status of Juvenile Sea Bass Fed on Diets Including Ulva Previously Treated or Not with a Mixture of Carbohydrases

A first point to consider in the evaluation of different types of seaweeds as ingredients in fish feeds is the level of inclusion. In this sense, positive effects on weight gain, specific growth rate, and feed use efficiency have been notified for most species when rates are less than 10% of the dry weight of the feed [4,5,39,40]. In contrast, levels of around 10% do not determine changes in the mentioned parameters [6,7,8,41], but higher amounts of 20% or more negatively affect growth parameters [7,9,10,41]. Considering all these preliminary results, in the present work two levels of dietary inclusion, i.e., 5% and 8%, were evaluated.

The first observed result after the inclusion of *U. ohnoi* meal in the feeds was a modification in the potential bioaccessibility of some readily soluble nutrients. The significant reduction in the amount of soluble protein in diets, including the seaweed, when compared to the control could be related to the presence of complex polysaccharides, which may represent more than 50% of its composition [42,43]. These molecules could interfere with the release of soluble protein carried out in an aqueous medium and also modify the rates of hydrolysis of the whole digesta. On the other hand, the significant reduction in soluble protein associated with the enzyme treatment of the seaweed meal could be explained considering that commercial products are obtained from the fermentation of fungi or yeasts with different degrees of purification. Therefore, although they are enriched in a series of main enzymatic activities (carbohydrases in this case), they can also have residual proteases and lipases [44], the former acting as hydrolyzing agents with an effect on the soluble protein fraction. On the other hand, no significant increase in the amount of reducing sugars or pentoses was evidenced, suggesting that the potential effect of the enzyme mixture could result in hydrolysis, mostly rendering oligosaccharides and not the above-indicated smaller compounds. The content of potential bioactives, measured as total phenols or TEAC, was significantly higher in the diet, including *U. ohnoi* when compared to those of the control diet, and no negative effect of the enzyme treatment was evidenced. This suggests that if the intended use of seaweeds as *U. ohnoi* is focused as sources of both nutrients and bioactive ingredients, an enzyme pretreatment could be carried out without affecting the potential effectiveness of these latter.

In a similar way to what was described by several authors [45,46], the enzymatic pretreatment of the seaweed meal was oriented to increase the bioavailability of nutrients by partially hydrolyzing the polysaccharide matrix. In the present study, such a positive effect was neither evidenced by changes in the bioavailability of key compounds (soluble protein, sugars, phenols) nor in the nutritive use of the feeds. The limited efficiency of the enzyme treatment was confirmed by the scanning images that evidenced the maintenance of the integrity of tissues to a great extent (Figure 2). It can be suggested that more effective hydrolysis could have been carried out using a different enzymatic compound to that used in the present study (Rovabio Max L) since its combination of enzymes was specifically designed to hydrolyze the fraction of non-starch polysaccharides present in terrestrial plants, but not in seaweeds such as *Ulva*. In spite of containing a significant fraction of xylan [47,48] potentially susceptible to hydrolysis, its high contents in the sulfopolysaccharide ulvan seem to make it especially resistant to chemical rupture [49]. In fact, enzymatic hydrolysis of this compound without including an aggressive phase of acid treatment requires specific enzymes (ulvan-lyases or ulvanases) identified to date only in some bacteria of marine origin [50,51].

The reported effects of the inclusion of *Ulva* in feeds for aquaculture are highly conditioned by the amount of algae used, the specific species of fish, and the species of *Ulva*. In the case of sea bass, reported results are contradictory. The inclusion of up to 10% of *Ulva lactuca* in feeds containing 65% fishmeal used for early juveniles (0.22 g average weight) produced results equivalent to those obtained with the control feed [4]. In contrast, the inclusion of 5–10% of *Ulva rigida* in a feed with 60% fishmeal for juveniles (4.7 g of average weight) determined a decrease in rates of growth and feed use [5]. Peixoto et al. (2016) [52] reported better growth rates, weight, and food use in fish of 24 g of average weight when including up to 7.5% of an unidentified *Ulva* meal in a feed with 30% fishmeal and a greater variety of ingredients. Results obtained in the present work when including 5% or 8% of *U. ohnoi* meal evidenced no significant effect on growth or feed efficiency, so they would be in line with the first two studies and not so much with the last one. However, it must be considered that the huge variability in the composition in macro- and micronutrients of the different *Ulva* species may greatly influence these results. Even results obtained when using the same species of algae in different species may also be variable. In the specific case of *U. ohnoi* used in the present work, its inclusion at 5%, despite having some positive effects on the integrity of the intestinal epithelium, impaired growth rates, and feed efficiency when included in feeds for *Solea senegalensis* [53], and hence limited use of this alga in feeds is recommended for this species. In contrast, an equivalent level of inclusion in a feed for *Salmo salar* did not produce significant differences in growth or feed use [54], although in this case, the evaluation was not carried out with a raw algal meal but using a derived product (Verdemin©), which could potentially have different physical-chemical and nutritional characteristics.

Few studies provide data on protein digestibility in feeds, including *Ulva*, but these point out important species-related differences. Norambuena et al. (2015) [54] reported a negative effect of *Ulva* on protein digestibility and suggested this could be determined by the limited capacity in the hydrolysis of complex polysaccharides. Nevertheless, after considering that the inclusion of *Ulva* represented only 1–2% of the total protein in their diets, the authors suggested that the reduction in protein digestibility could be due to a negative interaction between some components of the products of algae and proteolytic enzymes. In contrast, values of protein digestibility for juvenile sea bass obtained in the present study were not negatively affected by the presence of *U. ohnoi*, regardless of the level or enzyme treatment, this supporting the previously indicated negligible effect of compounds that could negatively affect the activity of enzymes. This should be in line with results reported by Valente et al. (2006) [5] when testing 5–10% *Ulva rigida* in sea bass feeds, suggesting that in this species, the digestive capacity is sufficient to adequately hydrolyze these amounts of algae and could also explain why no significant effect was obtained from the enzyme pretreatment.

Immunomodulation has been observed in many studies using seaweed extracts. Although the actual phytoimmunostimulant compounds are unknown, some studies suggest that polysaccharides present in seaweeds may activate the non-specific immune responses in both teleost and shrimps [55], and they may be more effective in enhancing mucosal immunity than systemic immunity [56]. Nevertheless, results obtained with different species may be somewhat contradictory. As an example, the use of a seaweed mix including *Fucus sp., Gracilaria sp.* and *Ulva sp.* as a supplement in diets for European sea bass, subjected to either combined salinity and temperature oscillations, did not mitigate the negative effects of such environmental changes on growth performance and innate immune responses [18]. In addition, lack of skin and gill mucosal immune stimulation has been reported when testing a 5% dietary inclusion of *U. ohnoi* in diets for *S. senegalensis* [19], but the authors suggest it could be due to the low inclusion level used. In contrast, supplementation with 5% *Ulva* spp. increased resistance to infection by *Pasteurela piscicida* in red seabream [57] and extracts obtained from *Ulva spp*. and *Chondrus crispus* have shown to increase respiratory burst and immune system stimulation in turbot and Atlantic salmon phagocytes [58]. In the present study, a significant increase in the activity of mucus lysozyme and alkaline phosphatase was evidenced in sea bass fed on the higher level of *U. ohnoi*. This result is in line with the increase in plasma lysozyme described in other species fed on feeds supplemented with different seaweeds such as kelp (*Ecklonia cava*) in olive flounder [53], *Gracilaria sp* in sea bass [54], or *Ulva* in seabream [50].

### 4.3. Experiment 3. Evaluation of the Effect of U. ohnoi on the Oxidative Status of Fish Challenged by the Consumption of a Feed Including Rancid Oil

The initial characterization performed in Experiment 2 of the present study (Table 2) indicated that although the inclusion of *U. ohnoi* in diets did not increase significantly the contents of total phenolic compounds when compared to a control diet, they determined a significant increase in Trolox equivalent antioxidant capacity (TEAC). For this reason, a specific experiment was designed to assess the potential protective effect of *U. ohnoi* against oxidative stress derived from intake of feeds, including rancid oil, using, in this case, a different species (gilthead seabream) than that used in previous experiments.

Surprisingly, no negative effects on food acceptance or growth performance were observed in fish fed on feeds, including this altered oil. The present results are similar to those reported in this same species by Mourente et al. (2002) [59] and by other authors in different species such as European sea bass *D. labrax* [60], Atlantic halibut (*Hippoglossus hippoglossus*) [61], or the Chinese longsnout catfish (*Tachysurus dumerilii)* [62]. The lack of response in growth and feed intake in the present study suggests that the species apparently is not very sensitive to oxidized fish oil, or maybe the experimental duration was not long enough to obtain an effect in such parameters. Nevertheless, metabolic indicators pointed to internal effects not readily evidenced as growth responses. After the initial period of challenge, values of HSI indicated a significantly smaller size of livers in fish fed on diets including *Ulva* and also a decrease in the case of fish initially fed on the control diet with rancid oil after the recovery period. This suggests changes in hepatic lipid metabolism that determined a lower deposit or higher mobilization of certain lipid classes associated with the consumption of *Ulva*. The same lowering effect on HSI has been previously reported in European seabass fed with *Gracilaria gracilis* supplemented diets at 8% [63]. This fact can be associated with reduced total lipids content, including triglycerides or cholesterol both in plasma or in the liver, as previously demonstrated in red sea bream (*Pagrus major*) fed *Spirulina sp*., which may reflect a high activity of key enzymes related to fatty acid β-oxidation to activate lipid mobilization [64]. Moreover, controverted or not so clear results have been reported in the evaluation of hepatosomatic index as a mirror of energetic balance mainly related to lipid metabolism in different fish cultured species after different challenges [65,66,67], so the protective role of this seaweed regardless of the state of the oil present in aquafeeds could not be ruled out.

On the other hand, changes in the immunological and oxidative status were evidenced in the present study. A decreased activity of mucus lysozyme was associated with the consumption of rancid oil, but normal levels seemed to be restored during recovery. This result should be in line with the increased activity of this enzyme measured in sea bass associated with the consumption of feeds, including *U.* *ohnoi* in Experiment 2. Regarding the oxidative status, it is clear that variations in the different parameters evaluated (lipid peroxidation and antioxidant enzymes) were influenced to a different extent either by the consumption of rancid oil, by the presence of *Ulva* in the diet, or by the interaction between them. Various studies have indicated that the extracts derived from seaweeds and microalgae are natural sources of antioxidants that neutralize free radicals [68]. SOD and CAT are important enzymes in the antioxidant defense system, as they play a key role in removing free radicals and toxicity of drugs and chemicals [69]. In the present study, the values of MDA evidenced the effect of consuming rancid oil in any of the two feeds (C or U) during the initial challenge period, being in agreement with results obtained in studies with other species [70,71]. The important point is that such values were reverted during the recovery period, but only in those fish that initially have received a feed, including *Ulva*. This suggests that some compounds present in the seaweed may exert a hepatoprotective role that enhances the active metabolism of oxidized lipids that could be accumulated in the liver [72].

The observed reduction in the activity of liver GPx associated with the consumption of *U. ohnoi* is in agreement with results reported when including *Gracilaria sp*. in diets for the same species [73] even at lower levels (2.5%). In addition, high values of CAT associated with the inclusion of seaweeds in diets have been described in a number of studies [67,74,75]. Again, the observed reduction in the activity of CAT in fish initially fed on any of the diets, including rancid oil (UO, CO), that after receiving the feed with *U. ohnoi* during the recovery period suggests the onset of a metabolic response oriented to reduce the amount of oxidation products. A similar effect was reported by Tocher et al. (2003) [71] in different marine fish such as turbot *S. maximus*, halibut (*H. hippoglossus*), and gilthead sea bream (*S. aurata*) fed on rancid oil when received supplementation of vitamin E, suggesting a protective role against oxidative metabolic misbalances. In addition, the reduced (although not significant) activity of SOD measured in the present study in fish receiving the U diet should be in line with results reported by Safavi et al. (2019) [76], who found significantly lower levels of this enzyme in livers of rainbow trout (*Oncorhynchus mykiss*) fed on extracted polysaccharides from *Ulva* and *Gracillaria.*

## 5. Resume and Conclusions

From all the previously described experiments, it can be concluded that:

- Contrarily to previous reports, no significant negative effect on protein digestion could be expected when using *U. ohnoi* as an ingredient in feeds when included at levels usually used for this kind of products (10% of the diet or less);
- *U. ohnoi* meal presents a reduced value as a nutritional ingredient when used in aquafeeds, even after being enzymatically treated to partially hydrolyze its carbohydrate fraction. Nevertheless, different results could be obtained if enzymes specifically capable of hydrolyzing ulvan could be used;
- In contrast to the above-mentioned, the role of *U. ohnoi* meal as a source of bioactive compounds is confirmed. An immunostimulatory effect was evidenced by an increase in mucus lysozyme in two different species (sea bass and sea bream). In addition, some compounds present in *U.* *ohnoi* perhaps positively influence the oxidative metabolism of the fish, being able to counteract the negative effects resulting from acute stress produced by the consumption of rancid oil.

## Figures and Tables

**Table 1:** Ingredients and proximal composition of the five feeds used in Experiment 2. The number (5, 8) indicates the % inclusion of the *Ulva ohnoi* meal, while the enzyme treatment is indicated by “E”. (*) Gross energy was estimated from nutrient analysis.

Ingredients (g/100 g)	C	U5/UE5	U8/UE8
Fish meal	32.0	32.0	32.0
Soybean meal	8.0	8.2	8.7
Guar meal	10.0	6.0	5.0
Soy concentrate	20.0	20.2	21.1
Corn gluten	15.0	15.0	16.0
Dried *Ulva* meal	0.0	5.0	8.0
Wheat meal	3.7	0.5	0.0
Fish oil	4.9	4.9	5.0
Sunflower oil	3.9	4.0	4.0
Soy lecithin	0.4	0.4	0.4
Vit/min premix	0.5	0.5	0.5
Taurine	0.5	0.5	0.5
Palatability enhancer	0.1	0.1	0.1
Cr_2_O_3_	1.0	1.0	1.0
Crude protein (%)	52.0	51.6	50.8
Fat (%)	14.0	14.2	14.0
Ash (%)	7.0	7.2	7.4
Moisture (%)	10.6	11.2	11.0
Gross energy (MJ/kg) *	20.8	20.9	20.6

**Table 2:** Ingredients and proximal composition of the four feeds used in Experiment 3. (*) Gross energy was estimated from nutrient analysis.

Ingredients (g/100 g)	C/CO	U/UO
Fish meal	25.0	25.0
Soybean meal	13.3	13.1
Guar meal	10.0	10.0
Soy concentrate	10.0	10.0
Corn gluten	15.0	15.0
Dried *Ulva* meal	0.0	10.0
Defatted rice bran	8.0	0.0
Wheat starch	5.3	3.3
Fish oil (oxidized or not)	6.1	6.2
Sunflower oil (oxidized or not)	4.9	5.0
Soy lecithin	1.2	1.2
Vit/min premix	0.8	0.8
Taurine	0.2	0.2
Attractant	0.2	0.2
Crude protein (%)	45.0	44.8
Fat (%)	12.1	12.4
Ash (%)	7.42	8.03
Moisture (%)	8.12	8.77
Gross energy (MJ/kg) *	19.7	19.8

**Table 3:** Experiment 2. Nutrient content of the feeds expressed in g/100 d.m. Values are presented as mean ± SD. Values in columns not sharing the same letter differ significantly with *p* < 0.05. Comparisons between inclusion levels (0%, 5%, 8%) are indicated with capital letters, while paired comparisons between enzyme treatment (U5/UE5; U8/UE8) are indicated with lowercase letters.

Feed	Soluble Protein	Reducing Sugars	Pentoses	Totals Phenols	TEAC (mM)
C	2.16 ± 0.25 ^A^	0.43 ± 0.09 ^A^	0.30 ± 0.04	0.13 ± 0.01	0.47 ± 0.03 ^A^
U5	0.87 ± 0.10 ^Ba^	0.25 ± 0.04 ^Ba^	0.32 ± 0.06	0.13 ± 0.01	1.02 ± 0.03 ^B^
U8	0.60 ± 0.03 ^Ca^	0.20 ± 0.01 ^B^	0.32 ± 0.05	0.12 ± 0.01	0.97 ± 0.01 ^B^
UE5	0.62 ± 0.01 ^b^	0.16 ± 0.02 ^b^	0.40 ± 0.04	0.14 ± 0.03	0.94 ± 0.01
UE8	0.13 ± 0.05 ^b^	0.28 ± 0.01	0.37 ± 0.04	0.12 ± 0.01	1.01 ± 0.00

**Table 4:** Experiment 2. Growth and efficiency in the use of food in experimental feeds. Values are presented as mean ± SD. Values in a row not sharing the same letter are significantly different, with *p* < 0.05. FCR: feed conversion ratio; SGR: specific growth rate; ADC: apparent digestibility coefficient.

Parameter	C	U5	UE5	U8	UE8
Initial weight (g)	508.37 ± 0.89	507.69 ± 6.07	510.22 ± 3.43	512.72 ± 1.43	508.05 ± 4.57
Final weight (g)	1265.35 ± 52.72	1261.73 ± 27.67	1249.66 ± 23.29	1217.24 ± 39.55	1195.20 ± 12.79
Weigh increase	756.98 ± 52.45	754.05 ± 25.07	739.43 ± 26.71	704.52 ± 39.34	687.16 ± 17.00
Feed intake (g)	998.45 ± 46.46	1005.59 ± 14.41	1007.84 ± 7.77	964.41 ± 12.97	945.72 ± 8.04
FCR	1.31 ± 0.06	1.32 ± 0.03	1.35 ± 0.05	1.37 ± 0.06	1.37 ± 0.03
SGR (% day^−1^)	1.38 ± 0.04 ^a^	1.38 ± 0.04 ^a^	1.35 ± 0.02 ^a^	1.29 ± 0.05 ^b^	1.29 ± 0.02 ^b^
ADC Protein	82.79 ± 0.03 ^a^	81.76 ± 0.52 ^a^	84.92 ± 0.52 ^b^	84.30 ± 0.87 ^b^	84.86 ± 0.33 ^b^

**Table 5:** Experiment 2. Activities of mucosal enzymes (in U/mg protein) measured in juveniles of *D. labrax* fed on diets including different amounts of *U. ohnoi* meal. Values presented as mean ± SD. Values in a row not sharing the same letter differ significantly with *p* < 0.05.

	C	U5	U8
Lysozyme	52.99 ± 18.33 ^a^	37.81 ± 5.23 ^a^	82.75 ± 30.01 ^b^
Alkaline phosphatase	2,376.13 ± 428.48 ^a^	2,798.81 ± 718.83 ^ab^	3,221.11 ± 857.98 ^b^

**Table 6:** Experiment 3. Bioactive compounds content of the experimental feeds expressed in g/100 m.s. Values presented as mean ± SD. Values in a column not sharing the same letter differ significantly with *p* < 0.05.

Feed	Total Phenols	TEAC (mM)
C	0.79 ± 0.08	0.87 ± 0.00 ^a^
CO	0.85 ± 0.05	0.88 ± 0.02
U	0.81 ± 0.08	0.92 ± 0.00 ^b^
UO	0.85 ± 0.09	0.92 ± 0.03

**Table 7:** Experiment 3. Biometric parameters obtained after the two phases of the experiment. Values presented as mean ± SD. Values in a column not sharing the same letter differ significantly with *p* < 0.05.

Parameter	C	CO	U	UO
Initial weight (g)		46.31 ± 0.29		
Challenge				
Final weight (g)	57.55 ± 2.05	56.95 ± 3.45	57.39 ± 4.43	59.32 ± 6.59
HSI (%)	1.21 ± 0.15 ^a^	1.28 ± 0.10 ^a^	1.11 ± 0.11 ^b^	1.11 ± 0.12 ^b^
Recovery				
Final weight (g)	62.84 ± 2.70	63.25 ± 2.23	61.27 ± 3.73	64.64 ± 6.14
HSI (%)	1.31 ± 0.22 ^a^	1.18 ± 0.12 ^a^	1.10 ± 0.04 ^b^	1.08 ± 0.10 ^b^

**Table 8:** Experiment 3. Immunological and oxidative status measured in mucus and liver as a response to oxidation associated with the consumption of a feed enriched with macroalgae. Comparisons of enzyme activities between feeds within each stage (challenge/recovery) are indicated with capital letters, while those made between each feeding phase for the same diet are detailed with a small letter. Values presented as mean ± SD. Values not sharing the same superscript differ significantly with *p* < 0.05.

	C	CO	U	UO
Challenge				
*In mucus*				
Lysozyme (U/mg protein)	50.81 ± 14.35 ^A^	38.25 ± 3.67 ^Ba^	48.28 ± 7.66 ^AB^	44.12 ± 4.17 ^AB^
*In liver*				
GPx (U/mg protein)	75.08 ± 5.55 ^A^	65.98 ± 648 ^AB^	54.41 ± 11.93 ^C^	57.10 ± 11.79 ^BC^
SOD (U/mg protein)	44.96 ± 7.51	43.91 ± 6.40	38.28 ± 3.64	44.96 ± 2.46
CAT (U/mg protein)	246.72 ± 1.23 ^A^	580.92 ± 31.25 ^BCa^	541.45 ± 32.58 ^B^	626.55 ± 59.57 ^C^
MDA (nmol/mg protein)	77.72 ± 15.40 ^A^	144.60 ± 67.62 ^B^	97.55 ± 30.26 ^A^	111.71 ± 15.54 ^ABa^
Recovery				
*In mucus*				
Lysozyme (U/mg protein)	50.81 ± 10.21	59.89 ± 15.96 ^b^	48.28 ± 23.40	55.77 ± 18.47
*In liver*				
GPx (U/mg protein)	75.08 ± 15.34 ^A^	76.70 ± 15.58 ^A^	54.41 ± 14.16 ^C^	61.25 ± 12.47 ^AB^
SOD (U/mg protein)	44.96 ± 2.82	44.88 ± 8.14	38.28 ± 7.49	58.56 ± 25.02
CAT (U/mg protein)	246.72 ± 51.44 ^A^	333.67 ± 66.25 ^Ab^	541.45 ± 80.04 ^B^	529.70 ± 93.31 ^B^
MDA (nmol/mg protein)	77.72 ± 11.86 ^B^	188.18 ± 35.66 ^C^	97.55 ± 32.56 ^B^	42.58 ± 10.35 ^Ab^

## Data Availability

Data available on request from the authors.
